# Types of social networks and starting leisure activities in later life: A longitudinal Japan Gerontological Evaluation Study (JAGES)

**DOI:** 10.1371/journal.pone.0254828

**Published:** 2021-07-15

**Authors:** Takuya Sekiguchi, Katsunori Kondo, Mihoko Otake-Matsuura

**Affiliations:** 1 Center for Advanced Intelligence Project, RIKEN, Chuo-ku, Tokyo, Japan; 2 Department of Social Preventive Medical Sciences, Center for Preventive Medical Sciences, Chiba University, Chiba, Chiba, Japan; 3 Department of Gerontological Evaluation, Center for Gerontology and Social Science, National Center for Geriatrics and Gerontology, Obu, Aichi, Japan; Sun Yat-sen University, CHINA

## Abstract

Considering beneficial effects of leisure activities in later life on well-being and health, we investigated which type of social network among older adults is associated with starting their participation in leisure activities. We used data from a longitudinal Japan Gerontological Evaluation Study (JAGES) conducted in Japan every three years from 2010 to 2016. We extracted types of social networks of older adults who did not participate in leisure activities in 2013 and responded to items related to social networks (n = 3436) relying on latent class analysis to examine changes in leisure activity participation over a three-year period within each latent class while controlling for participants’ activity in 2010. As a result, we identified five latent classes of social networks: the Neighborhood network, the Restricted network, which is characterized by limited social contacts, the Colleagues network, the Same-Interest network, and the Diverse network, from the most to the least prevalent. We found that members of the Neighborhood (Cohen’s *d* = 0.161) and Same-Interest networks (*d* = 0.660) were significantly more likely to, and members of the Diverse (*d* = 0.124) and Colleague networks (*d* = 0.060) were not significantly more likely to start leisure activities than those in the Restricted network. Furthermore, we found that lower age, better mental health, and higher education level were positively associated with starting participation in leisure activities in some latent classes. Horticulture or gardening was most likely to be chosen across all latent classes. Supporting the formation of social networks facilitating leisure activities, and recommending activities that were likely to be selected could be one solution for getting and keeping older adults active.

## Introduction

Leisure activities have been found to be a significant component of later life in terms of subjective well-being [1–6], reduced risk of cognitive decline and dementia [7–10], functional decline [11], and survival benefits [5, 12–14]. While the social relationships formed or strengthened by leisure activities may affect health, the leisure activities themselves can also have a positive effect on health. In fact, the positive association between even playing a game alone and cognitive function has been found [15].

Although the health benefits of leisure activities still require further investigations, several explanations have been proposed (for reviews, see [8, 16, 17]). For example, a cognitive stimulation can increase cognitive reserve [18], and that can delay the clinical onset of dementia. In addition to being a benefit in itself, improved mental health, which has been reported to be positively associated with leisure activities [1], may reduce the risk of developing various diseases caused by mental ill-health, such as cardiovascular diseases as argued in [8].

Given that leisure activities generally have positive effects, it is worthwhile to examine the association between the attributes of older adults and participation in leisure activities. Although overall decline in leisure participation with age has often been observed, a number of studies have shown that the likelihood of engagement in leisure activities differs depending on not just age, but also other factors such as gender; education status; social networks; region; mental and physical health; and type of activity [19–25]. This implies that it is possible to increase the likelihood of participating in leisure activities even in late life, if certain conditions are satisfied.

Previous literature has shown that participation in leisure activities is compatible with experience in earlier life. Ref. [26] showed that adulthood participation (not limited to older adults) in some kinds of activities is related to experience of these activities in childhood. Ref. [27] reported that older gardeners tended to have started gardening when they were young. Those results imply the possibility that health outcomes in later life examined in the aforementioned studies may actually be caused by activities in their youth, rather than later life. As Ref. [12] pointed out, longitudinal studies that differentiate the beneficial effects of leisure activities in later life from those in earlier life could serve as more cogent evidence and rationale for encouraging inactive older adults to participate in leisure activities.

Some studies focusing on changes in leisure activities using longitudinal data [9, 12] have indeed shown the beneficial effects of late-life leisure activities on health as distinguished from earlier activities. Ref. [12] reported that the significant association between late-life participation in some leisure activities and survival benefits remained even after controlling for earlier leisure participation and health status. Ref. [9] found a lower risk of dementia in older adults who start leisure and social activities, as well as in those who continue such activities from an early age.

Despite the benefits of late-life participation in leisure activities, as shown in studies that attempt to detect intrapersonal patterns of change in leisure participation [20, 28], having the cross-sectional limitation, older adults who cease participation in an activity tend not to start a new one, although the likelihood of their ceasing an activity declines with age. It is worth asking therefore what factors encourage people to start activities in later life.

The study of non-participation in leisure activities has clarified the barriers to participation from various perspectives ([29–31] for reviews, and see [32–36] for efforts to refine frameworks and concepts) although the scope of those studies is not always limited to older adults. Previous studies (e.g., [37]) have shown that such barriers to leisure participation are due to a variety of factors. Ref. [38] showed that older adults were more likely to belong to clusters characterized by the combination of constraints such as cost or lack of transportation, no opportunity near home, lack of information about where to learn or participate in an activity, and lack of partners.

One possible factor that could remove or alleviate these barriers is the social network in which older adults are embedded, which is compatible with the concept of the interpersonal barrier/facilitator in the framework of the research on (non-)participation in leisure [34, 36, 38]. Social networks have a variety of functions such as social influence, social support, and companionship [39], each of which may promote leisure activities. For example, informational support, which is a type of social support, may encourage older adults to take interest in activities they know little about. Companionship, defined as “the sharing leisure or other activities with network members” [39], can resolve the problem of a lack of partners. In fact, there have been empirical studies that are related to this theory. The number of friends and opportunities of being invited to participate were shown to be positively associated with social activities [40]. Ref. [41] demonstrated that leisure activities mediate the association between social relationships, which were defined by social support and strain, and better physical and psychological health.

According to the continuity theory [42, 43], older adults choose their lifestyle based on their past experiences, social relationships, and preferences, in the process of adaptation to changes in later life circumstances. Relying on this theory, it is reasonable to predict that intrapersonal patterns of change in leisure activities vary depending on social networks formed in earlier adulthood.

Although study participants were not limited to older adults, the result in [44] lends partial support to our hypothesis: the richness of social networks, which was assessed by the frequency and quality of contacts, could have a positive effect on the higher level of leisure-time physical activities even after controlling for age; however, the significance of this association varies depending on type of social network (relative, friend, or neighbor). Meanwhile, Ref. [45] showed that the degree of positive and negative social influences on physical activity of older adults varied depending on these influences’ sources (family, friend, or expert).

The association between participants’ social networks and leisure activities at a single time point has often been shown in prior studies. The following questions are therefore still open: What types of social networks facilitate intrapersonal patterns of changes in leisure participations, namely, starting participation in leisure activities in later life? Does a significant predictor of starting leisure activities differ across different types of social networks? These questions are important to understand how the environment may be improved to encourage older adults to participate in leisure activities even in late life based on the characteristics of each type of social network.

In previous studies, a lower number of activities were associated with adverse health outcomes. In Ref. [7], participants engaged in no leisure activities had a higher incidence of dementia than those who participated in a higher number of activities. Along this line, we aim to extract a typology of social networks among older adults not engaged in any leisure activities at baseline, and to find social networks encouraging those most inactive people to start an activity at follow-up.

To assess the total impact of social networks on starting activities, we characterized social networks, not based on their specific functions, but simply in terms of frequencies of social contacts and diversity of members. The question of which sociodemographic and health-related characteristics of older adults are significant predictors of starting leisure activities within each type of social network was also examined.

## Materials and methods

### Data set

Data from the Japan Gerontological Evaluation Study (JAGES), a nation-wide longitudinal self-reported questionnaire survey to understand social determinants of the health of community-dwelling independent older adults in Japan [46, 47] were used. The survey included over 100 questions about participants’ health, psychological, and functional status, and social relations and environment. The questionnaire explicitly stated that the participation was voluntary and the obtained responses would be used for research. JAGES interpreted their returns of the questionnaire as implying consent.

**Fig 1:**
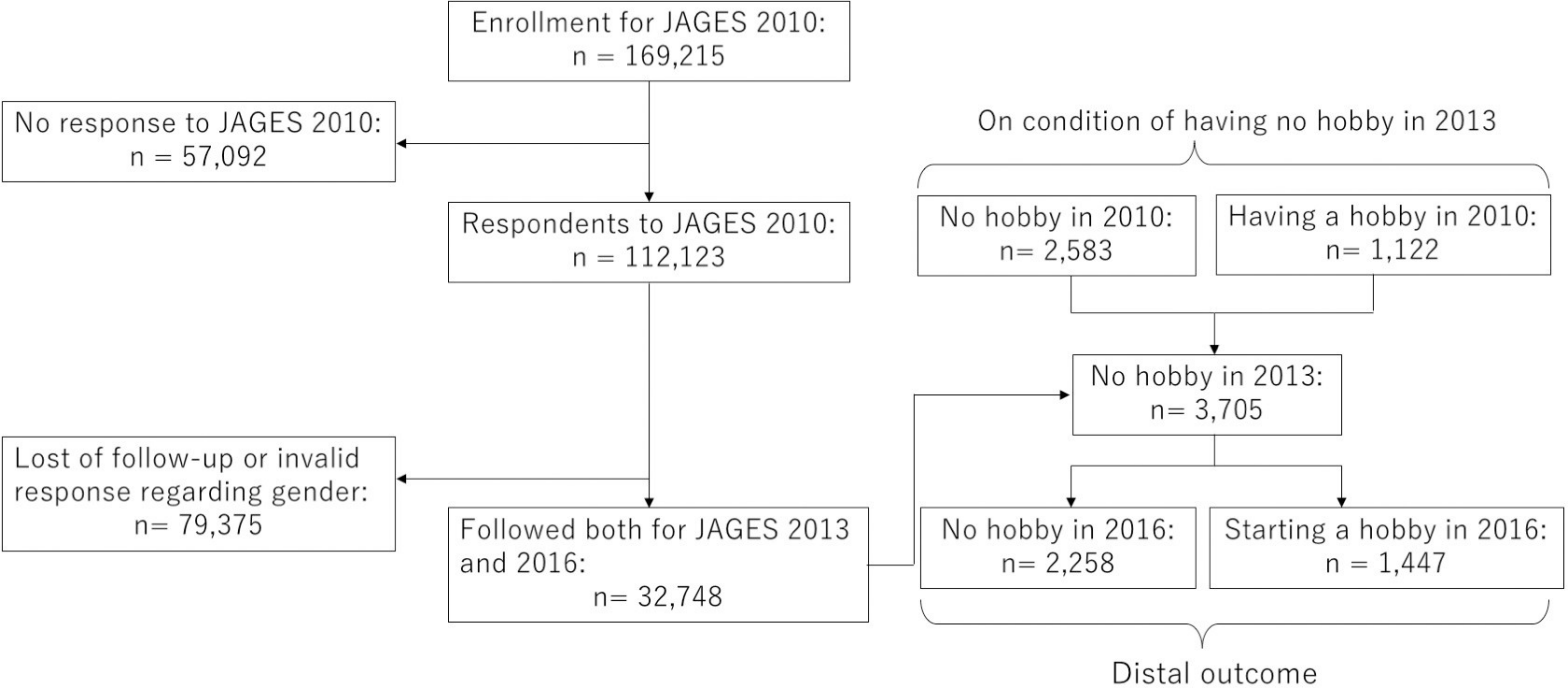
Flowchart of participants.

The 2010 wave was conducted from August 2010 through January 2012 on older adults aged 65 years and over who did not receive long-term care. A total of 169,215 people were randomly sampled from 31 municipalities, and 112,123 out of these returned the questionnaire (response rate: 66.3%). The 2010 wave was followed by the 2013 and 2016 waves, and the survey is still ongoing. We used the data set “panel10_13_16 ver. 1.0.,” which included participants who responded to the 2010, 2013, and 2016 waves and resided in the municipalities with available data on all three waves to construct the study panel data. Participants with loss of follow-up or invalid responses regarding gender were excluded. The resulting sample size and number of municipalities were 32,748 and 19, respectively (Fig 1).

The question of whether participants had hobbies, which are assumed to be one type of leisure activity in this paper (see the argument in the Distal outcome section), had been included in all three waves. However, the list of specific activities from which participants were asked to choose in 2010 was different from that in the 2013 and 2016 waves, although activities in the latter two were identical. Nevertheless, correspondence of the elements of the list in 2010 with those in 2013 and 2016 is proposed by the JAGES. We performed analyses according to the correspondence when tracking changes in specific hobby activities.

This study was approved by ethics committees at Nihon Fukushi University (Nos. 10–05 and 13–14), National Centre for Geriatrics and Gerontology (No. 992), and Chiba University (No. 2493).

### Distal outcome

Participants were first asked to respond to the question of whether they had hobbies, following which they were asked to choose their hobbies from a list of the following 20 hobbies: ground golf, golf, *pachinko* (Japanese pinball), calisthenics or *tai chi chuan*, walking or jogging, using the computer, reading, go (or *shogi* or *majan*), painting, fishing, karaoke, dancing, handicrafts, calligraphy, Japanese tea ceremony or flower arrangement, growing crops, horticulture or gardening, photography, travelling, and others. We defined a participant who chose at least one hobby as a participant with a hobby. To find factors associated with starting activities, whether participants had a hobby in 2016 on the condition that they had no hobby in 2013 was treated as the distal outcome of our latent class analysis (whether participants had a hobby in 2010 was used as the covariate as we will argue later in the Covariates section). For this analysis, we used the data on 3705 of 32,748 participants who had no hobbies in 2013 (Fig 1).

We describe the characteristics of hobby activities and the outcome of our analysis in comparison with similar concepts: social participation, social activities, and leisure activities. Although these concepts are not always clearly defined or classified, such conceptualization may be useful for future studies that examine in detail what aspects of specific activities are associated with health outcomes.

Leisure activities are often defined as unpaid work, not to maintain a household. Social participation and/or social activities, which overlap with leisure activities in some respects, can include paid work in a broad definition [48] and is often defined in terms of participation in a group, interactions with others, and its influence on and contributions to society (e.g., [49]). Indeed, the JAGES questionnaire has another question asking about social participation, and the activities listed therein are of that nature (e.g., senior citizen club, activities to support parents raising children, and local living arrangement improvement activities). Meanwhile, a hobby is one type of leisure activity in the sense that it is unpaid and not for a household, but its defining characteristic is enjoyment, and does not necessarily imply participation in a group or social impact, unlike social participation. The distinction between the concepts of leisure and hobby activities is often ambiguous. In order not to lose the connection with previous studies, we thus basically use the word "hobby" for discussions specific to the current analysis, and the word "leisure" for more general discussions. Nevertheless, it should be noted that the present study may be partially incompatible with future studies on the health effects of a stimulating social environment resulting from challenging social issues.

Furthermore, the items related to hobbies in the JAGES questionnaire were formulated based on free answers from a 2006 survey by JAGES’ predecessor, AGES (Aichi Gerontological Evaluation Study). The listed activities are therefore recognized as hobbies by older Japanese adults, which they choose to do often.

We focus on the likelihood of older adults starting any hobby, regardless of the hobby type. Nevertheless, it is expected that the magnitude of association with social networks varies depending on the hobby type. Our list of hobbies includes both social and solitary ones. However, as we have already argued, the functions of social networks are diverse (including sharing information about community activities, frequent invitations to participate in hobby activities, and information exchange, etc.), and the mechanism of starting leisure activities presumed by this study was not limited to engaging in activities with friends. Therefore, the magnitude of association between starting activities and social networks should be explained by the extent to which the activities coexist with rich social networks, rather than whether the activities are social or solitary.

For the reason, the same analyses were also conducted for starting hobbies that were found to be significantly associated with richness of social networks in the cross-sectional study by Ref. [50] and for those that were not, which we hereafter refer to as Types A and B, respectively. The resultant classification is as follows: ground golf, calisthenics, computer, reading, dancing, calligraphy, growing crops, gardening, photography, and travelling are Type A activities while golf, pachinko, walking, go, painting, fishing, karaoke, handcrafts, and tea ceremony are Type B activities (see S1 Table for the summary of Ref. [50] and the classification procedure details). The associated results will be presented in the Impact of different outcomes section.

### Latent class analysis indicators

Indicators pertaining to social networks consisted of the following eight questionnaire items: (Q1) how often participants met friends/acquaintances, (Q2) how many friends/acquaintances they had met over the past month, and (Q3) which types of people they met often: (i) neighbors, (ii) childhood friends, (iii) school friends, (iv) colleagues or former colleagues, (v) friends with the same interest or leisure activity, and/or (vi) friends in the same volunteer activity. The following responses to the question on the frequency of meeting friends (Q1), “Four or more times a week,” “Two or three times a week,” “Once a week,” or “One to three times a month” were recoded as “1,” and “a few times a year” or “rarely” were recoded as “0.” Responses to the question on the number of friends (Q2) were dichotomized into “10 or more” (= 1) and its complement (= 0). Each of the remaining questions Q3i–Q3vi had binary response alternatives (yes = 1/no = 0).

Although the indicator “friends with the same interest or leisure activity” seems inconsistent with the definition of not having a hobby, we included it because the word “same interest” in the item may, in principle, be different from hobby activities, and also because such social relationships may have been formed before 2013. Our data showed that among participants having no hobby in 2013 but in contact with friends with the same-interest in 2013 (n = 249), 47.0% (n = 117) had often met friends with the same-interest in 2010 as well. Moreover, 38.2% (n = 95) were engaged in hobby activities in addition to the contact in 2010. That is, even though our study participants had not engaged in hobby activities in 2013, some of them had maintained contact with friends with the same hobbies or interest in 2013. It is quite possible that information and invitations received from previously formed networks could influence starting activities, which is within the scope of the present study’s interest. However, our data cannot determine whether the person whom they had contacted was the same person in 2010 and 2013. Even in that case, at least, the tendency to meet people with the same interest itself continues, which may influence starting activities.

### Covariates

We used the following sociodemographic, and mental and physical health variables measured in 2013 as covariates: participants’ age, gender, education level, equivalent income, whether they were living alone, depressive symptoms assessed by the short version of the Geriatric Depression Scale (GDS-15) with 15 equally weighted items [51], and instrumental activities of daily living (IADL) measured by the Rouken-Shiki Scale [52] with 5 equally weighted items.

The JAGES data set contains self-reported ages and genders. Missing responses were compensated by JAGES based on information registered in lists of participants from the studied municipalities. We used the latter. For the Education variable, responses “10 to 12 years” or “13 years or more” to the question “How many years of formal education have you had?” were categorized as “1,” or “0” for other responses indicating fewer years. To equivalize income, we first calculated the middle number of the range given in each response option to the question “What was your pre-tax annual household income for 2012 (including pension)?” (e.g., for the response option “2.5 million to less than 3 million yen,” 2.75 million yen was used). This value was then divided by the square root of the number of household members. Some participants responded that the number of household members were zero even though the corresponding question was “How many people are in your household, *including yourself*?” We interpreted such responses to mean that the participant lived alone.

For the classification of social networks, it is promising to treat the living alone variable as the indicator variable. However, we found that that treatment did not affect the number of latent classes and that the conditional item-response probability pertaining to this variable within each class was not different enough to characterize each class. Therefore, we decided to use the living alone variable as the covariate, which was expected to clarify how the interrelation between each type of friend network and family connection affects starting activities, which was particularly examined in the Class-varying slopes section.

For GDS-15 scores, we used 4/5 as cut-off. A score of 5 to 15 points indicates mild to severe depression [53]. Participants were divided according to their IADL scores into whether or not they got the perfect score (5 points) based on the findings that a variation of 1 point in the IADL score indicates an unignorable change in IADL performance [54]. Missing values for Education, Equivalent income, GDS, and IADL were estimated using stochastic regression imputation [55].

In addition to socio-demographic and health factors, having a hobby in 2010 (Fig 1) was also included. This allowed us to estimate the effects of other variables on starting hobby activities over a three-year period from 2013–2016 while controlling for participants’ activity three years before the baseline.

### Statistical analysis

The chi-squared test and the *t*-test were performed for descriptive statistics in the two groups of participants without a hobby both in 2013 and 2016 and those who had had no hobbies in 2013 but had started hobby activities in 2016 to compare differences in characteristics between the two groups.

Applying latent class analysis with auxiliary variables, we estimated the number of latent classes (Class enumeration section), the degree to which covariates were associated with each latent class membership by regressing estimated latent class membership on covariates (Latent class regression section), and the degree to which associations between covariates and changes in hobby activities differ across latent classes (Class-varying slopes section). Using the logistic regression models in which the participants’ most likely latent class is an independent variable, we also examined which latent class members were more likely to start hobby activities (Main effect of latent class membership section).

For the latent class analysis with auxiliary variables, we adopted the following three-step approach [56, 57]. This approach first applies latent class analysis to the set of indicator variables without a distal outcome and covariates. In this step, the number of latent classes are determined, and a posterior probability distribution where each participant belongs to each latent class is generated. In the second step, classification uncertainty, which indicates the degree of unreliability of the participants’ most likely latent class membership, is calculated based on the posterior distribution generated in the first step. In the final step, latent class analysis of the set of indicator variables with a distal outcome and covariates is performed by using the most likely latent class membership with the classification uncertainty rates obtained in the second step. This approach is expected to mitigate bias due to ignorance about the imprecision of assigning latent class membership.

In our latent class analysis, full information maximum likelihood estimation was used to handle missing data on indicator variables. As a result, 269 participants with missing data on all indicator variables were excluded. To determine the number of latent classes, the Bayesian Information Criterion (BIC) was referred to for information of model fit evaluation.

In addition to the analyses of overall hobby activities, we investigated which specific hobby activities were likely to be selected within each estimated latent class (Specific hobby activities section). Moreover, we also examined how much participants within each latent class were likely to resume the specific activities they engaged in in 2010. Hereafter, we use the term “*resume*” when indicating that participants had the same hobby in 2010 and 2016, “*restarting*” when they had a hobby in 2010 and 2016 regardless of the type of hobby, and “*starting*” when they had a hobby in 2016 regardless of their activity status in 2010.

In addition to the main analysis tracking changes in hobby activities from 2013 to 2016, we presented a cross-sectional analysis to examine the association between having hobbies in 2013 and the study explanatory variables as Supporting Information: descriptive statistics (S2 Table) and results of the logistic regressions (S3 Table) by using the whole sample in order to clarify the characteristics of participants inactive in 2013.

Mplus version 8.1 [58] was used for latent class analysis, and R version 3.4.3 [59] was used for all other statistical analyses.

## Results

### Preliminary characterization of the participant subgroup

This study extracts the latent class of participants having no hobby in 2013. We here characterize the focal participants having no hobby in 2013 by the result of the cross-sectional analysis of factors associated with having a hobby in 2013 for the whole sample (S2 and S3 Tables).

The cross-sectional analysis shows that most indicator variables regarding social networks (except for Childhood friend variable) are significantly positively associated with having a hobby cross-sectionally, even after controlling for activity status in 2010 (S3 Table). This means that the latent classes extracted in the subsequent analysis are conditional on being in a group of people with weak social ties.

### Descriptive statistics

Frequencies of the indicators, covariates, and distal outcomes stratified by participant subgroups of those who did not have a hobby in 2016 and those who started hobby activities in 2016 are provided in Table 1. We observed that starting participation in activities from the baseline in 2013 was relatively unlikely to occur. Table 1 also shows that participants’ characteristics were significantly associated with starting hobby activities in 2016, although their effect sizes were small: Those who started activities were likely to have high contact with friends and a larger number of friends, often meet colleagues or former colleagues, friends with the same interest or leisure activities, and friends with the same volunteer activities, be younger, more highly educated, and less depressed, and have higher IADL scores. Those who started activities significantly more likely to have at least one hobby in 2010, with the highest effect size.

**Table 1:** Frequency distributions of the distal outcome, indicators, and covariates.

	Distal outcome		Effect size	*p* value
	Inactive (*n* = 2258)	Starting (*n* = 1447)		
	*n* (%)	*n* (%)		
Indicators				
Q1: Frequency (≥1–3 times monthly)	1132 (50.1)	825 (57.0)	0.088	< 0.001
Missing	234 (10.4)	174 (12.0)	0.026	0.128
Q2: Number of friends (≥ 10)	335 (14.8)	311 (21.5)	0.096	< 0.001
Missing	243 (10.8)	169 (11.7)	0.014	0.416
Q3i: Neighbor	1180 (52.3)	802 (55.4)	0.033	0.062
Q3ii: Childhood friend	197 (8.7)	115 (7.9)	0.015	0.421
Q3iii: Friend from their school days	240 (10.6)	185 (12.8)	0.034	0.053
Q3iv: Colleague or former colleague	536 (23.7)	396 (27.4)	0.043	0.015
Q3v: Friend with the same interest	84 (3.7)	165 (11.4)	0.158	< 0.001
Q3vi: Friend in the same volunteer activity	32 (1.4)	59 (4.1)	0.088	< 0.001
Missing (Q3i–Q3vi)	227 (10.1)	141 (9.7)	0.005	0.802
Covariates				
Age (mean ± SD)	75.39 ± 5.55	74.55 ± 5.16	0.155	< 0.001
Gender = female	1399 (62.0)	851 (58.8)	0.031	0.060
Equivalent income (mean ± SD)	189.45±125.64	194.89±128.25	0.043	0.204
Education years ≥ 10	853 (37.8)	606 (41.9)	0.041	0.014
GDS≥5	1242 (55.0)	671 (46.4)	0.084	< 0.001
IADL<5	744 (32.9)	395 (27.3)	0.060	< 0.001
Living alone	280 (12.4)	156 (10.8)	0.025	0.150
Active in 2010	497 (22.0)	625 (43.2)	0.225	< 0.001

*Note*. SD = standard deviation; GDS = Geriatric Depression Scale; IADL = instrumental activities of daily living. Missing values in covariates were imputed by stochastic regressions. The chi-squared test was used for categorical variables and the *t*-test for continuous variables. The associated effect sizes for categorical and continuous variables were measured using Cramer’s *V* and Cohen’s *d*, respectively.

### Class enumeration

We selected the five-class model as it had the lowest BIC value. Table 2 gives the information used to determine the number of latent classes.

**Table 2:** Model fit information for determining the number of latent classes.

Classes	LL	AIC	BIC	aBIC	Entropy	VLRT	BLRT
1	-11699.756	23415.511	23464.648	23439.228			
2	-11373.959	22781.917	22886.332	22832.315	0.794	0.000	0.000
3	-11282.041	22616.081	22775.775	22693.160	0.558	0.001	0.000
4	-11239.242	22763.455	22763.455	22652.244	0.591	0.000	0.000
5	-11185.902	22459.804	22730.055	22590.246	0.765	0.001	0.000
6	-11154.485	22414.971	22740.500	22572.094	0.646	0.002	0.000

*Note*. LL = log likelihood; AIC = Akaike’s Information Criterion; BIC = Bayesian Information Criterion; aBIC = adjusted BIC; VLRT = p-value for the Vuong-Lo-Mendell-Rubin Likelihood Ratio Test; BLRT = approximate p-value for the Bootstrap Likelihood Ratio Test.

### Class characterization

#### Item-response probabilities within latent classes

The extracted latent classes were labeled based on the item-response probabilities for each latent class (Table 3). To name latent classes, we partially referred to previous studies on social network types [60]. The “Neighborhood network” (41.1%) was the most prevalent class. Members in this class tended to have a high frequency of meeting friends and meet neighbors frequently. The “Restricted network” (26.5%) was the second largest class, and was characterized by low frequency of meetings and a small number of friends being met. The “Colleague network” (20.2%) was the third most prevalent class and was characterized by a moderate frequency of meeting friends. Members in this class tended to meet colleagues or former colleagues and not meet neighbors. The “Diverse network” (3.2%) and “Same-Interest network” (9.1%) were less prevalent, and characterized by a higher frequency of meeting friends. Members in the “Diverse network” class were likely to meet all types of friends except friends with the same interest or leisure activity. The “Same-Interest network” was characterized by the highest likelihood of seeing friends with the same interest or leisure activities, as well as the highest probability of frequent contact.

**Table 3:** Conditional item-response probabilities within the five latent classes.

	Restricted	Diverse	Same-Interest	Neighbor	Colleague
Class prevalence	.265	.032	.091	.411	.202
Indicators					
Q1: Frequency	.000	.850	.978	.844	.646
Q2: Number	.000	.584	.400	.251	.180
Q3i: Neighbor	.388	1.00	.511	1.00	.000
Q3ii: Childhood friend	.037	.631	.077	.070	.136
Q3iii: Friend from their school days	.073	.811	.087	.087	.189
Q3iv: Colleague or former colleague	.156	.537	.248	.215	.541
Q3v: Friend with the same interest or leisure activity	.028	.098	.697	.000	.000
Q3vi: Friend in the same volunteer activity	.004	.080	.075	.028	.025

#### Latent class regression

To determine the characteristics of each latent class based on study covariates, our latent class model with auxiliary variables comprised multinomial logistic regression analysis of latent class membership (outcome) and covariates (explanatory variables).

Table 4 shows the odds ratio of each latent class membership, using the Restricted network as the reference category. Participants having a higher GDS score and lower IADL functionality were significantly more likely to populate the Restricted network than any of the remaining networks except the Same-Interest network for IADL. Female participants were significantly more likely to belong to the Same-Interest and Neighborhood networks. Age was the factor associated with a reduced likelihood of belonging to the Same-Interest and Colleague networks. Participants with higher education were significantly less likely to belong to the Neighborhood network. Higher income significantly increased the likelihood of belonging to the Diverse network. Having at least one hobby in 2010 significantly increased the likelihood of belonging to the Same-Interest network. Living alone significantly increased the likelihood of belonging to the Same-Interest, Neighborhood, and Colleague networks.

**Table 4:** Latent class regression coefficients in the five-class model.

	Diverse	Same-Interest	Neighborhood	Colleague
	OR (95% CI, *p*)	OR (95% CI, *p*)	OR (95% CI, *p*)	OR (95% CI, *p*)
Age (continuous)	0.970 (0.922–1.021, 0.249)	0.957** (0.926–0.989, 0.009)	1.000 (0.979–1.022, 0.964)	0.956** (0.928–0.985, 0.003)
Gender = female (ref. male)	1.363 (0.725–2.563, 0.337)	1.929*** (1.353–2.750, 0.000)	2.522*** (1.986–3.203, 0.000)	1.075 (0.796–1.450, 0.639)
Equivalent income (continuous in units of one million yen)	1.200* (1.018–1.414, 0.031)	1.020 (0.896–1.161, 0.758)	0.969 (0.870–1.079, 0.558)	1.120 (0.986–1.272, 0.084)
Education years ≥ 10 (ref. < 10)	1.406 (0.750–2.638, 0.287)	0.986 (0.696–1.398, 0.937)	0.687** (0.542–0.870, 0.002)	0.926 (0.694–1.235, 0.601)
GDS ≥ 5 (ref. < 5)	0.426** (0.231–0.786, 0.006)	0.341*** (0.240–0.484, 0.000)	0.534*** (0.420–0.680, 0.000)	0.705* (0.524–0.950, 0.022)
IADL< 5 (ref. = 5)	0.364* (0.156–0.851, 0.020)	0.800 (0.548–1.168, 0.247)	0.650** (0.509–0.830, 0.001)	0.658** (0.481–0.900, 0.009)
Living alone (ref. Living with at least one family member)	1.815 (0.728–4.524, 0.200)	2.740*** (1.598–4.697, 0.000)	1.704** (1.116–2.602, 0.014)	2.081** (1.238–3.499, 0.006)
Active in 2010 (ref. Inactive)	1.826 (0.958–3.479, 0.068)	5.743*** (4.020–8.205, 0.000)	1.232 (0.955–1.590, 0.109)	1.194 (0.864–1.649, 0.285)

*Note*. Restricted network is the reference class. OR = odds ratio; CI = confidential interval; ref. = reference; GDS = Geriatric Depression Scale; IADL = instrumental activities of daily living.

* *p* < 0.05;

** *p* < 0.01;

*** *p* < 0.001.

### Main effect of latent class membership

The logistic regression model was used to determine the likelihood of starting hobby activities, which differed by latent class; the Restricted network class was used as the reference category.

Table 5 shows that the membership of the Same-Interest (OR = 3.313) and Neighborhood networks (OR = 1.340) significantly increased the likelihood of starting hobby activities in 2016 compared to membership of the Restricted network. In particular, the Same-Interest network membership had a large effect size. Members of the Colleague (OR = 1.155) and the Diverse networks (OR = 1.252) were not significantly more likely to start hobby activities.

**Table 5:** Result of the logistic regression analysis predicting the likelihood of starting activities (outcome: 1 = starting activities; 0 = remaining inactive).

	OR	95% CI	Cohen’s *d*	*p* value
Latent class				
Diverse (*n* = 84, 35/49)	1.252	0.786–1.994	0.124	0.344
Same-Interest (*n* = 223, 153/70)	3.313***	2.402–4.568	0.660	< 0.001
Neighbor (*n* = 1360, 538/822)	1.340***	1.129–1.591	0.161	< 0.001
Colleague (*n* = 575, 217/358)	1.155	0.932–1.433	0.060	0.188
Covariates				
Age (continuous)	0.969***	0.956–0.983	0.017	< 0.001
Gender (ref. male)	0.930	0.797–1.086	0.040	0.359
Income (continuous in units of one million yen)	1.003	0.946–1.063	0.002	0.924
Education years ≥ 10 (ref. < 10)	1.117	0.960–1.301	0.061	0.153
GDS ≥ 5 (ref. < 5)	0.765***	0.661–0.886	0.148	< 0.001
IADL < 5 (ref. = 5)	0.833*	0.705–0.984	0.101	0.031
Living alone (ref. Living with at least one family member)	0.816	0.648–1.029	0.112	0.086
Active in 2010 (ref. Inactive)	2.475***	2.119–2.891	0.500	< 0.001

*Note*. Restricted Network (*n* = 1194, 384/810) is the reference class. This analysis was conducted after assigning participants to their most likely latent class. The first element in parentheses shown after each latent class indicates the number of assigned participants. The numerator (respectively, denominator) of the second element is the number of participants starting activities (respectively, remaining inactive).

OR = odds ratio; CI = confidential interval; GDS = Geriatric Depression Scale; IADL = instrumental activities of daily living.

* *p* < 0.05;

*** *p* < 0.001.

Table 5 also shows that participants who were younger, with a lower GDS score, with higher IADL functionality, and a hobby in 2010 were significantly more likely to start hobby activities.

### Class-varying slopes

How much the likelihood of starting activities is differentially associated with covariates across latent classes was also simultaneously estimated in the latent class analysis with auxiliary variables.

Table 6 shows that within the Restricted network, a lower GDS score was significantly associated with a higher likelihood of starting hobby activities. Moreover, it was found that being younger within the Neighborhood and Colleague networks significantly increased the likelihood of starting hobby activities. Within the Diverse network, no covariates were significant predicting factors for starting hobby activities. Gender was not a significant predictor within any of the latent classes. Higher education level was a significant positive predictor within the Colleague network. Having a hobby in 2010 was a significant positive predictor within all classes except the Diverse network. It is noteworthy that the estimated odds ratio was notably higher in the Same-Interest network.

**Table 6:** Class-varying slopes associated with covariates (outcome: 1 = starting activities; 0 = remaining inactive).

	Restricted	Diverse	Same-Interest	Neighbor	Colleague
	OR (95% CI, *p*)	OR (95% CI, *p*)	OR (95% CI, *p*)	OR (95% CI, *p*)	OR (95% CI, *p*)
Age (continuous)	0.981 (0.948–1.014, 0.250)	0.928 (0.789–1.093, 0.371)	0.973 (0.909–1.042, 0.432)	0.965** (0.941–0.989, 0.004)	0.960* (0.923–0.999, 0.046)
Gender = female (ref. male)	0.816 (0.555–1.198, 0.299)	0.976 (0.249–3.829, 0.972)	0.716 (0.349–1.472, 0.364)	0.926 (0.692–1.239, 0.604)	1.045 (0.682–1.600, 0.841)
Equivalent income (continuous in units of one million yen)	1.135 (0.983–1.311, 0.084)	1.164 (0.842–1.61, 0.357)	1.095 (0.788–1.521, 0.590)	0.944 (0.842–1.058, 0.323)	0.889 (0.762–1.036, 0.131)
Education years ≥ 10 (ref. < 10)	1.201 (0.816–1.769, 0.353)	0.666 (0.183–2.427, 0.538)	1.098 (0.563–2.142, 0.785)	0.963 (0.734–1.263, 0.783)	1.557* (1.006–2.408, 0.047)
GDS ≥ 5 (ref. < 5)	0.543** (0.368–0.802, 0.002)	0.571 (0.148–2.201, 0.416)	0.614 (0.311–1.213, 0.160)	1.003 (0.778–1.293, 0.980)	0.845 (0.563–1.268, 0.416)
IADL < 5 (ref. = 5)	0.725 (0.487–1.079, 0.113)	0.457 (0.078–2.696, 0.387)	0.959 (0.462–1.991, 0.912)	0.950 (0.707–1.277, 0.733)	0.783 (0.483–1.270, 0.322)
Living alone (ref. Living with at least one family member)	1.593 (0.688–3.686, 0.277)	1.413 (0.237–8.443, 0.705)	0.760 (0.319–1.810, 0.535)	0.731 (0.491–1.088, 0.122)	0.506 (0.255–1.002, 0.051)
Active in 2010 (ref. Inactive)	2.640*** (1.736–4.016, 0.000)	2.475 (0.649–9.433, 0.184)	5.024*** (2.556–9.876, 0.000)	2.220*** (1.678–2.939, 0.000)	1.750**(1.108–2.764, 0.016)

*Note*. OR = odds ratio; CI = confidential interval; GDS = Geriatric Depression Scale; IADL = instrumental activities of daily living.

* *p* < 0.05;

** *p* < 0.01;

*** *p* < 0.001.

### Impact of different outcomes

The counterparts of the above results in the two analyses in which starting hobbies having association (i.e., Type A activities) and not having association (i.e., Type B activities) with social networks in the cross-sectional study by Ref. [50] were used as distal outcomes are presented in Tables 7–10.

**Table 7:** Frequency distributions of the distal outcomes Type A and B, indicators, and covariates.

	Distal outcome		Effect size	*p* value
	Remaining inactive	Starting activities		
	*n* = 2591	*n* = 1114		
	*n* = 2995	*n* = 710		
	*n* (%)	*n* (%)		
Indicators				
Q1: Frequency (≥1–3 times monthly)	1327 (51.2)	630 (56.6)	0.049	0.003
	1541 (51.5)	416 (58.6)	0.056	< 0.001
Missing	273 (10.5)	135 (12.1)	0.023	0.158
	331 (11.1)	77 (10.8)	0.003	0.874
Q2: Number of friends (≥ 10)	397 (15.2)	249 (24.7)	0.085	< 0.001
	493 (16.5)	153 (21.5)	0.053	0.001
Missing	285 (11.0)	127 (11.4)	0.006	0.722
	330 (11.0)	82 (11.5)	0.007	0.686
Q3i: Neighbor	1336 (51.6)	646 (58.0)	0.059	< 0.001
	1604 (53.6)	378 (53.2)	0.002	0.879
Q3ii: Childhood friend	224 (8.6)	88 (7.9)	0.012	0.453
	253 (8.4)	59 (8.3)	0.002	0.906
Q3iii: Friend from their school days	274 (10.6)	151 (13.6)	0.043	0.009
	332 (11.1)	93 (13.1)	0.025	0.130
Q3iv: Colleague or former colleague	617 (23.8)	315 (28.3)	0.047	0.004
	729 (24.3)	203 (28.6)	0.039	0.019
Q3v: Friend with the same interest or leisure activity	120 (4.6)	129 (11.6)	0.127	< 0.001
	147 (4.9)	102 (14.4)	0.149	< 0.001
Q3vi: Friend in the same volunteer activity	41 (1.6)	50 (4.5)	0.086	< 0.001
	58 (1.9)	33 (4.6)	0.069	< 0.001
Missing (Q3i–Q3vi)	264 (10.2)	104 (9.3)	0.013	0.426
	299 (10.0)	69 (9.7)	0.003	0.832
Covariates				
Age (mean ± SD)	75.26 ± 5.50	74.59 ± 5.17	0.124	0.001
	75.34 ± 5.54	73.90 ± 4.67	0.268	< 0.001
Gender = female	1597 (61.6)	653 (58.6)	0.028	0.084
	1862 (62.2)	388 (54.6)	0.061	< 0.001
Equivalent income including imputed values (mean ± SD)	189.11±124.85	197.30±130.71	0.065	0.071
	189.73±124.65	199.34±134.70	0.076	0.069
Education years ≥ 10 including imputed values	966 (37.3)	493 (44.3)	0.065	< 0.001
	1157 (38.6)	302 (42.5)	0.031	0.056
GDS≥5	1433 (55.3)	480 (43.1)	0.112	< 0.001
	1588 (53.0)	325 (45.8)	0.057	< 0.001
IADL<5	848 (32.7)	291 (26.1)	0.066	< 0.001
	954 (31.9)	185 (26.1)	0.049	0.003
Living alone	311 (12.0)	125 (11.2)	0.011	0.498
	358 (12.0)	78 (11.0)	0.012	0.472
Active in 2010	368 (12.1)	390 (31.4)	0.237	< 0.001
	344 (11.5)	255 (35.9)	0.261	< 0.001

*Note*. For each cell, the upper sub-cell represents the result when the distal outcome is defined as whether or not the Type A activity was started. The result in the lower sub-cell is the counterpart of the Type B activity.

SD = standard deviation; GDS = Geriatric Depression Scale; IADL = instrumental activities of daily living. Missing values in covariates were imputed by stochastic regressions. The chi-squared test was used for categorical variables and the *t*-test for continuous variables. The associated effect sizes for categorical and continuous variables were measured using Cramer’s *V* and Cohen’s *d*, respectively.

**Table 8:** Latent class regression coefficients in the five-class model in which Type A and B activities were used.

	Diverse	Same-Interest	Neighborhood	Colleague
	OR (95% CI, *p*)	OR (95% CI, *p*)	OR (95% CI, *p*)	OR (95% CI, *p*)
Age (continuous)	0.969 (0.920–1.022, 0.240)	0.960* (0.930–0.990, 0.011)	0.998 (0.977–1.020, 0.844)	0.953** (0.926–0.982, 0.002)
	0.971 (0.920–1.026, 0.299)	0.970 (0.940–1.001, 0.071)	0.999 (0.978–1.021, 0.933)	0.955** (0.927–0.984, 0.002)
Gender = female (ref. male)	1.305 (0.705–2.414, 0.398)	1.788** (1.256–2.544, 0.001)	2.540*** (1.996–3.232, 0.000)	1.087 (0.802–1.472, 0.590)
	1.213 (0.616–2.390, 0.576)	1.946*** (1.368–2.770, 0.000)	2.507*** (1.970–3.190, 0.000)	1.042 (0.772–1.406, 0.790)
Equivalent income (continuous in units of one million yen)	1.204* (1.026–1.414, 0.024)	1.023 (0.901–1.162, 0.727)	0.969 (0.871–1.077, 0.555)	1.115 (0.984–1.264, 0.089)
	1.195* (1.013–1.409, 0.035)	1.011 (0.890–1.148, 0.862)	0.955 (0.861–1.060, 0.383)	1.096 (0.969–1.240, 0.147)
Education years ≥ 10 (ref. < 10)	1.384 (0.738–2.596, 0.312)	0.977 (0.692–1.380, 0.897)	0.687** (0.541–0.873, 0.002)	0.932 (0.699–1.244, 0.634)
	1.412 (0.725–2.749, 0.311)	1.020 (0.724–1.438, 0.907)	0.682** (0.538–0.864, 0.002)	0.919 (0.690–1.223, 0.559)
GDS ≥ 5 (ref. < 5)	0.420** (0.224–0.786, 0.007)	0.352*** (0.249–0.499, 0.000)	0.530*** (0.416–0.676, 0.000)	0.692* (0.514–0.932, 0.016)
	0.420** (0.219–0.805, 0.009)	0.336*** (0.237–0.475, 0.000)	0.532*** (0.418–0.677, 0.000)	0.696* (0.518–0.933, 0.016)
IADL< 5 (ref. = 5)	0.361* (0.153–0.851, 0.020)	0.758 (0.520–1.104, 0.149)	0.657** (0.514–0.839, 0.001)	0.676* (0.494–0.926, 0.015)
	0.367* (0.148–0.910, 0.030)	0.825 (0.568–1.200, 0.314)	0.641*** (0.501–0.821, 0.000)	0.651** (0.475–0.893, 0.008)
Living alone (ref. Living with at least one family member)	1.896 (0.734–4.897, 0.186)	2.821*** (1.652–4.817, 0.000)	1.701* (1.109–2.607, 0.015)	2.090** (1.243–3.513, 0.006)
	1.696 (0.662–4.344, 0.272)	2.633*** (1.566–4.426, 0.000)	1.621* (1.070–2.456, 0.023)	1.944* (1.164–3.250, 0.011)
Active in 2010 (ref. Inactive)	1.677 (0.789–3.567, 0.179)	5.129*** (3.562–7.386, 0.000)	1.362* (1.005–1.846, 0.047)	1.260 (0.851–1.865, 0.248)
	1.306 (0.507–3.366, 0.581)	4.948*** (3.390–7.223, 0.000)	1.158 (0.827–1.623, 0.395)	1.020 (0.665–1.564, 0.928)

*Note*. Restricted network class is the reference class.

For each cell, the upper sub-cell represents the result when Type A activity was used. The result in the lower sub-cell is the counterpart of the Type B activity. OR = odds ratio; CI = confidential interval; ref. = reference; GDS = Geriatric Depression Scale; IADL = instrumental activities of daily living.

* *p* < 0.05;

** *p* < 0.01;

*** *p* < 0.001.

**Table 9:** Result of the logistic regression analysis predicting the likelihood of starting Type A and B leisure activities (outcome: 1 = starting a Type A activity (or B); 0 = otherwise).

	OR	95% CI	Cohen’s *d*	*p* value
Latent class				
Diverse (*n* = 84, 31/53; 15/69)	1.498	0.927–2.421	0.223	0.099
	1.027	0.563–1.873	0.015	0.930
Same-Interest (*n* = 223, 119/104; 91/132)	2.464***	1.801–3.371	0.497	< 0.001
	2.667***	1.909–3.726	0.541	< 0.001
Neighbor (*n* = 1360, 421/939; 257/1103)	1.320**	1.096–1.588	0.153	0.003
	1.287*	1.033–1.604	0.139	0.024
Colleague (*n* = 575, 161/414; 104/471)	1.073	0.850–1.356	0.039	0.553
	1.072	0.814–1.412	0.038	0.621
Covariate				
Age (continuous)	0.978**	0.963–0.992	0.012	0.003
	0.949***	0.931–0.967	0.029	< 0.001
Gender (ref. male)	0.897	0.761–1.057	0.060	0.194
	0.794*	0.655–0.962	0.127	0.019
Income (continuous in units of one million yen)	1.000	0.939–1.064	0.000	0.989
	1.028	0.957–1.104	0.015	0.452
Education years ≥ 10 (ref. < 10)	1.241**	1.056–1.459	0.119	0.009
	0.964	0.797–1.165	0.020	0.702
GDS ≥ 5 (ref. < 5)	0.663***	0.566–0.776	0.227	< 0.001
	0.855	0.711–1.028	0.086	0.095
IADL < 5 (ref. = 5)	0.769**	0.642–0.921	0.145	0.004
	0.836	0.677–1.033	0.099	0.097
Living alone (ref. Living with at least one family member)	0.928	0.727–1.186	0.041	0.550
	0.937	0.701–1.253	0.036	0.662
Active in 2010 (ref. Inactive)	3.014***	2.520–3.605	0.608	< 0.001
	3.813***	3.102–4.688	0.738	< 0.001

*Note*. Restricted Network (*n* = 1194, 290/904; 186/1008) is the reference class.

This analysis was conducted after assigning participants to their most likely latent class. The first element in parentheses shown after each latent class indicates the number of assigned participants. For each cell, the result when activity type A was used is shown in the upper sub-cell. The result in the lower sub-cell is the counterpart of the activity type B. The numerator (respectively, denominator) of the second element is the number of participants who started Type A activities (respectively, otherwise). The third element is the counterpart of Type B activities.

OR = odds ratio; CI = confidential interval; GDS = Geriatric Depression Scale; IADL = instrumental activities of daily living.

* *p* < 0.05;

** *p* < 0.01;

*** *p* < 0.001.

**Table 10:** Class-varying slopes associated with covariates when Type A and B activities were used (outcome: 1 = starting a Type A activity (or B); 0 = otherwise).

	Restricted	Diverse	Same-Interest	Neighbor	Colleague
	OR (95% CI, *p*)	OR (95% CI, *p*)	OR (95% CI, *p*)	OR (95% CI, *p*)	OR (95% CI, *p*)
Age (continuous)	0.999 (0.963–1.035, 0.942)	0.961 (0.829–1.113, 0.596)	0.992 (0.931–1.057, 0.802)	0.965* (0.939–0.991, 0.010)	0.961 (0.918–1.007, 0.097)
	0.960 (0.920–1.001, 0.050)	0.979 (0.762–1.258, 0.867)	1.001 (0.953–1.072, 0.718)	0.921*** (0.890–0.954, 0.000)	0.939** (0.895–0.985, 0.007)
Gender = female (ref. male)	0.874 (0.566–1.349, 0.543)	1.139 (0.271–4.782, 0.859)	0.644 (0.321–1.291, 0.215)	0.832 (0.610–1.135, 0.245)	1.052 (0.639–1.729, 0.843)
	0.720 (0.429–1.210, 0.142)	Not estimated due to no male living alone	0.534* (0.265–1.076, 0.015)	0.821 (0.564–1.193, 0.252)	0.819 (0.471–1.423, 0.432)
Equivalent income (continuous in units of one million yen)	1.115 (0.957–1.300, 0.163)	1.105 (0.841–1.452, 0.474)	1.213 (0.875–1.680, 0.246)	0.945 (0.834–1.072, 0.383)	0.845 (0.703–1.016, 0.074)
	1.190 (1.009–1.402, 0.057)	1.328* (1.050–1.680, 0.039)	1.050 (0.777–1.420, 0.755)	0.959 (0.826–1.114, 0.578)	0.845 (0.625–1.142, 0.234)
Education years ≥ 10 (ref. < 10)	1.295 (0.841–1.992, 0.240)	0.571 (0.157–2.074, 0.395)	0.967 (0.514–1.819, 0.916)	1.117 (0.836–1.491, 0.455)	1.999** (1.221–3.272, 0.006)
	0.968 (0.575–1.631, 0.901)	0.521 (0.059–4.594, 0.408)	1.537 (0.812–2.912, 0.283)	0.889 (0.623–1.269, 0.493)	0.903 (0.508–1.605, 0.713)
GDS ≥ 5 (ref. < 5)	0.574** (0.374–0.879, 0.011)	0.481 (0.121–1.913, 0.299)	0.576 (0.301–1.102, 0.096)	0.852 (0.649–1.118, 0.248)	0.581* (0.372–0.907, 0.017)
	0.533** (0.319–0.889, 0.001)	0.629 (0.032–12.194, 0.697)	0.941 (0.473–1.872, 0.857)	1.238 (0.878–1.746, 0.273)	0.838 (0.494–1.421, 0.473)
IADL < 5 (ref. = 5)	0.683 (0.442–1.057, 0.087)	0.328 (0.036–2.996, 0.324)	0.831 (0.408–1.691, 0.609)	1.001 (0.732–1.369, 0.997)	0.554* (0.310–0.991, 0.047)
	0.528** (0.304–0.917, 0.001)	1.975 (0.211–18.477, 0.665)	1.081 (0.519–2.252, 0.841)	0.630** (0.405–0.98, 0.009)	1.666 (0.934–2.972, 0.176)
Living alone (ref. Living with at least one family member)	1.631 (0.711–3.740, 0.248)	0.963 (0.114–8.158, 0.973)	1.373 (0.571–3.298, 0.479)	0.928 (0.601–1.432, 0.736)	0.41* (0.191–0.888, 0.024)
	2.027 (0.824–4.988, 0.270)	2.451 (0.232–25.870, 0.622)	0.617 (0.293–1.299, 0.102)	0.836 (0.494–1.416, 0.466)	0.605 (0.227–1.608, 0.190)
Active in 2010 (ref. Inactive)	3.236*** (1.906–5.496, 0.000)	1.834 (0.433–7.761, 0.410)	6.017*** (3.129–11.572, 0.000)	2.871*** (2.071–3.979, 0.000)	1.918* (1.043–3.527, 0.036)
	5.368** (3.003–9.593, 0.006)	5.208 (0.639–42.465, 0.450)	6.275** (3.335–11.808, 0.009)	3.628** (2.356–5.586, 0.001)	1.326 (0.552–3.189, 0.583)

*Note*. For each cell, the result when the distal outcome is defined as whether or not to start Type A activity is shown in the upper sub-cell. The result in the lower sub-cell is the counterpart of Type B activity. OR = odds ratio; CI = confidential interval; GDS = Geriatric Depression Scale; IADL = instrumental activities of daily living.

* *p* < 0.05;

** *p* < 0.01;

*** *p* < 0.001.

Table 7 reports descriptive statistics for both types of distal outcomes, which is the counterpart of Table 1. Some points found in Table 1 were retained: For both types, starting activities from the baseline in 2013 was relatively unlikely to occur. Furthermore, for both types, those who started activities were likely to have high contact with friends and a larger number of friends, often meet colleagues, friends with the same interest or leisure activities, and friends with the same volunteer activities, be younger and less depressed, and have higher IADL scores. Those who started activities significantly more likely to have at least one hobby in 2010, with the highest effect size. However, differently from Table 1, frequently meeting neighbors and friends from school days and higher education level were significantly more likely to be observed only for those who started Type A activities, and male, only for those who started Type B activities.

Table 8 shows the result of latent class regressions when using starting Type A and B activities as distal outcomes, which is the counterpart of Table 4. Comparison between Tables 4 and 8 showed almost no qualitative difference between them. Exceptionally, while age significantly increased the likelihood of belonging to the Restricted network than the Same-Interest network in Table 4, that was applicable only to Type A in Table 8. Furthermore, while being active in 2010 in terms of Type A activities was a significantly positive predictor to be in the Neighborhood network in Table 8, that was not in Table 4.

Table 9 shows the main effects of latent class memberships when using starting Type A and B activities as distal outcomes, which is the counterpart of Table 5. Comparison between Tables 5 and 9 showed no qualitative difference between them.

Table 10 shows the class-varying slopes associated with covariates when using starting Type A and B activities as distal outcomes, which is the counterpart of Table 6. Differently from the two comparisons above, the comparisons between Tables 6 and 10 revealed that slopes associated with the various covariates varied not only by latent class, but also by hobby type. In the Restricted network and the Neighborhood network, participants with lower IADL functionality were significantly less likely to start Type B activities while no association was found in Table 6. In the Diverse network, participants with higher income were significantly more likely to start Type B activities while no association was found in Table 6. In the Same-Interest network, male participants were significantly more likely to start Type B activities while no association was found in Table 6. In the Colleague network, higher education level, being active in 2010, and lower age were significantly positive predictors in Table 6, but in Table 10 the first two were associated only with starting Type A activities and the third one only with starting Type B activities. While in Table 6 participants with higher GDS scores, and lower IADL functionality were significantly less likely to start activities, that was applicable only to Type A activities. Living alone in the Colleague network was a significantly negative predictor of starting Type A activities while it was not in Table 6.

### Specific hobby activities

Frequencies of specific activities that participants started in 2016 within each latent class are shown in Table 11. Horticulture or gardening was found to be most likely to be chosen across all latent classes. Walking or jogging, reading, growing crops, and traveling were also relatively likely to be chosen across all latent classes.

**Table 11:** Distribution of participation in specific hobby activities within each latent class.

	Restricted (*n* = 1194)	Diverse (*n* = 84)	Same-Interest (*n* = 223)	Neighbor (*n* = 1360)	Colleague (*n* = 575)
	*n* (%)	*n* (%)	*n* (%)	*n* (%)	*n* (%)
Ground golf	9 (0.75)	1 (1.19)	10 (4.48)	28 (2.06)	11 (1.91)
Golf	5 (0.42)	0 (0.00)	8 (3.59)	6 (0.44)	1 (0.17)
Pachinko	13 (1.09)	1 (1.19)	9 (4.04)	20 (1.47)	15 (2.61)
Calisthenics	23 (1.93)	6 (7.14)	18 (8.07)	30 (2.21)	19 (3.30)
Walking	103 (8.63)	7 (8.33)	39 (17.49)	149 (10.96)	51 (8.87)
Computer	27 (2.26)	4 (4.76)	14 (6.28)	9 (0.66)	10 (1.74)
Reading	66 (5.53)	8 (9.52)	28 (12.56)	84 (6.18)	34 (5.91)
Go	17 (1.42)	0 (0.00)	14 (6.28)	8 (0.59)	6 (1.04)
Painting	4 (0.34)	0 (0.00)	8 (3.59)	12 (0.88)	5 (0.87)
Fishing	14 (1.17)	1 (1.19)	7 (3.14)	16 (1.18)	15 (2.61)
Karaoke	26 (2.18)	6 (7.14)	21 (9.42)	36 (2.65)	23 (4.00)
Dancing	3 (0.2)	1 (1.19)	8 (3.59)	4 (0.29)	1 (0.17)
Handicrafts	30 (2.51)	3 (3.57)	23 (10.31)	55 (4.04)	13 (2.26)
Calligraphy	6 (0.50)	0 (0.00)	3 (1.35)	10 (0.74)	3 (0.52)
Tea ceremony	2 (0.17)	0 (0.00)	5 (2.24)	8 (0.59)	0 (0.00)
Growing crops	78 (6.53)	10 (11.91)	39 (17.49)	166 (12.21)	45 (7.83)
Gardening	133 (11.14)	17 (20.24)	59 (26.46)	211 (15.51)	72 (12.52)
Photography	11 (0.92)	2 (2.38)	5 (2.24)	14 (1.03)	13 (2.26)
Travelling	52 (4.36)	9 (10.71)	36 (16.14)	90 (6.62)	47 (8.17)
Others	56 (4.69)	6 (7.14)	36 (16.14)	56 (4.12)	30 (5.22)

*Note*. This analysis was conducted after assigning participants to their most likely latent class.

S4 Table shows that among participants who had hobbies both in 2010 and 2013, only the Same-Interest network membership significantly increased the likelihood of resuming specific activities than the Restricted network membership.

## Discussion and implications

### Summary

Given previous literature showing that leisure activities, even if started in later life, are associated with health, it is of significance to examine the conditions under which inactive older adults are more likely to start activities. Social networks have a variety of functions that encourage older adults to be active, but their effect can vary depending on the type of network. To investigate this issue, we applied latent class analysis to social relationship characteristics of older adults having no hobbies (which were found to be characterized by relatively fewer social ties than people having hobbies as the cross-sectional analysis [S2 and S3 Tables] suggested), and examined which latent class memberships in 2013 were significantly associated with starting leisure activities over a three-year period from 2013–2016, while controlling for whether or not they had participated in leisure activities three years before the baseline (i.e., 2010).

As shown in Table 1 and Fig 1, the fraction of older adults having no hobby was low, and furthermore, they were less likely to start a hobby. The advantage of this study is that even such relatively rare events were statistically analyzed by using data with the large total sample size.

### Characteristics of the latent classes

By using latent class analysis, we extracted five latent classes of social relationships: the Neighborhood network, the Restricted network, the Colleague network, the Same-Interest network, and the Diverse network, ordered from most to least prevalent types (Table 3). Although the variable “friends with the same interest or leisure activity” has not necessarily been included in network classification studies, the present study demonstrated that the item-response probability of this indicator is not similar across latent classes, but rather that one independent class characterized by the high item-response probability of the indicator was extracted and associated with starting activities.

The latent class regression revealed that adverse health-related factors were associated with Restricted network membership (Table 4). Notably, participants living alone were more likely to belong to the Same-Interest, Neighborhood, and Colleague networks than the Restricted network. This can be interpreted to mean that these types of networks compensate for the lack of familial connections.

### Effect of each latent class membership

Further analysis revealed that members of the Neighborhood network and Same-Interest network were significantly more likely to start leisure activities in the next three years compared to those in the Restricted networks, whereas members of the Diverse network and Colleague network were not (Table 5). This result was not changed even when Type A and B activities were used as distal outcomes (Table 9).

Considering participants’ activities in 2010 is helpful to interpret the results regarding the Same-Interest network. Our analyses basically showed that those who had been active in 2010 were more likely to restart activities in 2016 (Active in 2010 variable in Tables 5 and 6). The Same-Interest network not only enhanced its magnitude more than other networks (Table 6), but also strengthened the tendency to resume specific activities (S4 Table). These results suggest that social relationships formed through leisure activities can later promote the resumption of leisure activities of older adults who were inactive for a while. As noted earlier, the rationale for this result could be described in relation to the continuity theory [42, 43]: resuming leisure activities is regarded as being an adaptive choice to changes of circumstances in later life, which are consistent with social relationships and activity preferences formed through leisure activities earlier and sustained across time. This result is also consistent with previous results that leisure activity participation earlier in life is significantly predictive of active participation in later life [61]. Since our study period is shorter than those of previous studies, future research into whether results of longer-term studies are consistent is needed.

For the Neighborhood network, sharing information about community activities and more frequent invitations to participate in hobby activities might lead to starting such activities [40].

Given the results regarding the Neighborhood, and Same-Interest networks, our findings suggest that there are several possible scenarios for intervention studies in which starting leisure activities is encouraged in older age.

On the one hand, the results regarding the Same-Interest network suggests that interventions specific to facilitating the building of networks of shared interests and hobbies may encourage older adults to resume previous activities that they stopped. On the other hand, interventions encouraging neighborhood relationships, even if not hobby-specific, may contribute to starting leisure activities as a spillover effect.

The Colleague network membership was not significantly more related to starting leisure activities than the Restricted network membership. One possible explanation could be that older adults who had lived far from their workplace and whose social network was mainly consisting of friends from work were likely to have less social interaction than those whose network consists of their neighbors, especially after retirement. Less social interaction and its resultant lack of information exchange could hinder them from starting leisure activities. The Colleague network had the second lowest item-response probabilities associated with contacts with friends and the number of friends (Table 3).

### Effect of each covariate within each latent class

Using class-varying slopes for covariates, we observed that the association between the likelihood of starting leisure activities and sociodemographic, mental, and physical factors varied across latent classes and types of activities (Tables 6 and 10). This means that when planning effective interventions or policies to facilitate activities among older adults, practitioners should consider the social networks in which older adults are embedded [60]; it is therefore informative to understand which covariates are predictive of being inactive or starting activities within a specific social network. For example, our results show that depression was associated with a lower likelihood of starting activities (Table 5) and depressed people were likely to belong to the Restricted network (Table 4). Besides, our result of class-varying slopes for covariates shows that members of the Restricted network were vulnerable to the effect of depression on starting activities (Table 6). Therefore, not only is it effective to prevent depressed older adults from becoming isolated and support forming other types of social networks than the Restricted network, it is also important not to exacerbate the depression of those who have become isolated in order to facilitate activities among them.

Table 10 revealed that the Colleague network was vulnerable to the effects of a variety of factors such as lower education level, higher GDS score, lower IADL functionality, and living alone on starting Type A activities. In terms of promoting leisure activities, the Colleague network probably should be given priority for intervention efforts, as should the Restricted network.

For factors that are difficult to change or inevitable, such as aging, in the cases of the Neighborhood and Colleague networks (Table 6), it may be beneficial to scrutinize which aspects of them reduce the likelihood of being active and find factors to compensate for this disadvantage. However, because the relationship between social network memberships and covariates is cross-sectional in the present study, future studies should clarify the direction of causality in the associations observed by using longitudinal data over more prolonged periods.

### Choices of specific activities

We found that the following specific activities were likely to be selected in all latent classes: walking or jogging, reading, growing crops, horticulture or gardening, and traveling (Table 11). Some studies have reported the association between participating in the above-listed leisure activities and beneficial outcomes. For example, physical activities, including walking, was associated with lower cognitive decline [62] and reduced risk of dementia [63]. Walking, gardening, and reading were positively associated with subjective well-being [3]. Ref. [64] found that older men who were active in growing crops, horticulture or gardening had a lower likelihood of being in long-term care with dementia. Positive associations between travel experiences among older adults and their quality of life [65, 66] or with self-rated health [67] have been reported. It is useful to identify which activities are not only beneficial for health reasons but also likely to be of interest in finding the solution for encouraging older adults’ to be active.

### Future studies

We suggest some major directions for further studies. The first promising research target is the classification of various patterns of starting hobby activities and the associations between each of those patterns and various health outcomes. Patterns here refer to the number and combination of, and level of engagement to hobbies. For example, Ref. [68] reported that the higher number of hobbies reduced the risk of dementia. It would be useful to examine which social networks can increase the number of hobbies being started. Ref. [27] found positive association between higher levels of older adults’ gardening engagement and various aspects of leisure satisfaction.

The interrelation between specific activities was not examined in this study, despite its importance. It will thus be fruitful to examine which activity is likely to trigger older adults to start other activities, and within which type of social network.

It is also promising to consider changes in social networks and health of older adults. Social network characteristics of older adults are typically not static, and these do not always change monotonically [69]. Moreover, some studies have demonstrated that health is associated with changes in social relations from midlife to late life [70] and in later life [71]. To answer the questions of whether and how the activities of older adults change in tandem with changes in social networks and how much such events lead to health benefits in late life will be a reasonable next step. Furthermore, analyses that include changes in sociodemographic and health-related characteristics [72] would also be useful.

## Conclusions

Social networks are important for older adults’ lives in terms of receiving social support, exchanging information, and companionship, which can motivate inactive older adults to start leisure activities. Our longitudinal study demonstrated this view, while also adding new perspectives as follows: the effect varies depending on the type of social network and activities; whether older adults are more likely to resume the same activity as before or start a new one depends on network types—although the scope was limited to three years before the baseline. However, the question remains as to which of the started activities were more likely to be sustained and in which social networks. This could be the focus of future longitudinal studies.

## Supporting information

S1 Table Summary of the results of Iwasa and Yoshida (2018), which led to the classification of hobby activities based on their relationship with the richness of social networks. (DOCX)

S2 Table Frequency distributions of the indicators and covariates in 2013 stratified by activity status in 2013. (DOCX)

S3 Table Result of the logistic regression analysis cross-sectionally predicting the likelihood of having hobby in 2013 (outcome: 1 = having hobby; 0 = having no hobby). (DOCX)

S4 Table Result of the logistic regression analysis predicting the likelihood of resuming specific leisure activities among participants who had hobbies both in 2010 and 2013 (outcome: 1 = resuming activities; 0 = starting new activities). (DOCX)
