# Supplementary material for: Types of social networks and starting leisure activities in later life: A longitudinal Japan Gerontological Evaluation Study (JAGES)
Source: PLoS One. 2021 Jul 15;16(7):e0254828. doi: 10.1371/journal.pone.0254828 (PMC8282000; doi:10.1371/journal.pone.0254828)
Supplement: S1 Table — (DOCX) [file pone.0254828.s001.docx]

**S1 Table. Summary of the results of Iwasa and Yoshida (2018), which led to the classification of hobby activities based on their relationship with the richness of social networks**

| Items in the JAGES data | Items in Iwasa & Yoshida (2018) | Factors in Iwasa & Yoshida (2018) | Type |
| --- | --- | --- | --- |
| Ground golf | Japanese croquet (ground golf) | Social-public | A |
| Golf | Recreational  (tennis, bowling, golf) | Competitive game | B |
| Pachinko | - | - | B |
| Calisthenics | Flexibility (stretching, yoga, tai chi); Weight lift, strength, calisthenics; Aerobics (cardio, fitness, workout) | Physical | A |
| Walking | Go for walks; Exercise (jog, bike, swim) | -; Physical | B |
| Computer | Use a computer | Technology use | A |
| Reading | Read for leisure | Development | A |
| Go | Play mah-jongg; Play go and shogi | Competitive game | B |
| Painting | Painting | Creative | B |
| Fishing | Outdoor (sail, fish, backpack) | - | B |
| Karaoke | Go to karaoke | - | B |
| Dancing | Japanese dance | Cultural | A |
| Handicrafts | Ceramic art | Creative | B |
| Calligraphy | Calligraphy | Cultural | A |
| Tea ceremony | Tea ceremony | Creative | B |
| Growing crops | Crop work | Raising plants | A |
| Gardening | Gardening | Raising plants | A |
| Photography | Engage in photography | Technology use | A |
| Travelling | Domestic travel; Travel abroad | Travel | A |

*Note*. In the analysis by Iwasa and Yoshida (2018), social networks were measured using the Japanese version of the Lubben Social Network Scale-6 (Kurimoto et al. 2011; Lubben et al. 2006). They applied factor analysis to 51 types of leisure activities. They excluded eight activities that did not belong to any factor (with factor loading < 0.3), and then implemented the factor analysis again. They regarded activities having factor loadings of 0.4 or higher in the later factor analysis as relevant ones. Subsequently, they examined associations between extracted factors and social networks. We defined activities as Type A if they had a factor loading of 0.4 or higher and had a statistically significant association with social networks; all others were classified as Type B.

**References**

Kurimoto A, Awata S, Ohkubo T, Tsubota-Utsugi M, Asayama K, Takahashi K, ... & Imai Y. Reliability and validity of the Japanese version of the abbreviated Lubben Social Network Scale. *Japanese Journal of Geriatrics*. 2011;48(2):149-157. doi:10.3143/geriatrics.48.149

Lubben J, Blozik E, Gillmann G, Iliffe S, von Renteln Kruse W, Beck J C, Stuck A E. Performance of an abbreviated version of the Lubben Social Network Scale among three European community-dwelling older adult populations. The Gerontologist, 2006;46(4):503-513. doi:10.1093/geront/46.4.503
