# Supplementary material for: Types of social networks and starting leisure activities in later life: A longitudinal Japan Gerontological Evaluation Study (JAGES)
Source: PLoS One. 2021 Jul 15;16(7):e0254828. doi: 10.1371/journal.pone.0254828 (PMC8282000; doi:10.1371/journal.pone.0254828)
Supplement: S2 Table — (DOCX) [file pone.0254828.s002.docx]

**S2 Table: Frequency distributions of the indicators and covariates in 2013 stratified by activity status in 2013**

|  | Status in 2013 | | Effect size | *p* value |
| --- | --- | --- | --- | --- |
|  | Inactive  (*n* = 3705) | Active  (*n* = 29043) |  |  |
|  | *n* (%) | *n* (%) |  |  |
| Indicators | | | | |
| Q1: Frequency (≥1−3 times monthly) | 1957 (52.8) | 21838 (75.2) | 0.159 | < 0.001 |
| Missing | 408 (11.0) | 1326 (4.6) | 0.091 | < 0.001 |
| Q2: Number of friends (≥ 10) | 646 (17.4) | 11503 (39.6) | 0.145 | < 0.001 |
| Missing | 412 (11.1) | 1151 (4.0) | 0.106 | < 0.001 |
| Q3i: Neighbor | 1982 (53.5) | 17412 (60.0) | 0.042 | < 0.001 |
| Q3ii: Childhood friend | 312 (8.4) | 3233 (11.1) | 0.028 | < 0.001 |
| Q3iii: Friend from their school days | 425 (11.5) | 5302 (18.3) | 0.057 | < 0.001 |
| Q3iv: Colleague or former colleague | 932 (25.2) | 9094 (31.3) | 0.042 | < 0.001 |
| Q3v: Friend with the same interest | 249 (6.7) | 13336 (45.9) | 0.252 | < 0.001 |
| Q3vi: Friend in the same volunteer activity | 91 (2.5) | 2512 (8.6) | 0.073 | < 0.001 |
| Missing (Q3i–Q3vi) | 368 (9.9) | 834 (2.9) | 0.119 | < 0.001 |
| Covariates | | | | |
| Age (mean±SD) | 75.06±5.41 | 74.3±4.97 | 0.147 | < 0.001 |
| Gender = female | 2250 (60.7) | 15770 (54.3) | 0.041 | < 0.001 |
| Equivalent income (mean±SD) | 191.57±126.68 | 225.50±137.41 | 0.257 | < 0.001 |
| Education years ≥ 10 | 1459 (39.4) | 16490 (56.8) | 0.111 | < 0.001 |
| GDS≥5 | 1913 (51.6) | 8275 (28.5) | 0.158 | < 0.001 |
| IADL<5 | 1139 (30.7) | 4444 (15.3) | 0.130 | < 0.001 |
| Living alone | 436 (11.8) | 3297 (11.4) | 0.004 | 0.470 |
| Active in 2010 | 1122 (30.3) | 23025 (79.3) | 0.353 | < 0.001 |

*Note*. SD = standard deviation; GDS = Geriatric Depression Scale; IADL = instrumental activities of daily living. Missing values in covariates were imputed by stochastic regressions. The chi-squared test was used for categorical variables and the *t*-test for continuous variables. The associated effect sizes for categorical and continuous variables were measured using Cramer’s *V* and Cohen’s *d*, respectively.
