# Supplementary material for: Types of social networks and starting leisure activities in later life: A longitudinal Japan Gerontological Evaluation Study (JAGES)
Source: PLoS One. 2021 Jul 15;16(7):e0254828. doi: 10.1371/journal.pone.0254828 (PMC8282000; doi:10.1371/journal.pone.0254828)
Supplement: S3 Table — (DOCX) [file pone.0254828.s003.docx]

**S3 Table: Result of the logistic regression analysis cross-sectionally predicting the likelihood of having hobby in 2013 (outcome: 1 = having hobby; 0 = having no hobby)**

|  | OR | 95% CI | Cohen’s *d* | *p* value |
| --- | --- | --- | --- | --- |
| Indicators | | | | |
| Q1: Frequency (≥1−3 times monthly) | 1.280^***^ | 1.189–1.372 | 0.136 | < 0.001 |
| Missing | 1.096 | 0.899–1.293 | 0.051 | 0.362 |
| Q2: Number of friends (≥ 10) | 1.250^***^ | 1.148–1.353 | 0.123 | < 0.001 |
| Missing | 1.026 | 0.816–1.235 | 0.014 | 0.812 |
| Q3i: Neighbor | 1.223^***^ | 1.135–1.311 | 0.111 | < 0.001 |
| Q3ii: Childhood friend | 1.104 | 0.968–1.240 | 0.055 | 0.153 |
| Q3iii: Friend from their school days | 1.254^***^ | 1.136–1.372 | 0.125 | < 0.001 |
| Q3iv: Colleague or former colleague | 1.207^***^ | 1.116–1.298 | 0.104 | < 0.001 |
| Q3v: Friend with the same interest | 5.226^***^ | 5.086–5.366 | 0.912 | < 0.001 |
| Q3vi: Friend in the same volunteer activity | 1.563^***^ | 1.336–1.789 | 0.246 | < 0.001 |
| Missing (Q3i–Q3vi) | 0.700^**^ | 0.475–0.926 | 0.196 | 0.002 |
| Covariates | | | | |
| Age (continuous) | 0.997 | 0.990–1.005 | 0.001 | 0.492 |
| Gender (ref. male) | 0.689^***^ | 0.605–0.772 | 0.206 | < 0.001 |
| Income (continuous in units of one million yen) | 1.008 | 0.977–1.039 | 0.005 | 0.609 |
| Education years ≥ 10 (ref. < 10) | 1.258^***^ | 1.177–1.340 | 0.127 | < 0.001 |
| GDS ≥ 5 (ref. < 5) | 0.597^***^ | 0.518–0.676 | 0.284 | < 0.001 |
| IADL < 5 (ref. = 5) | 0.573^***^ | 0.481–0.665 | 0.307 | < 0.001 |
| Living alone (ref. Living with at least one family member) | 0.887 | 0.765–1.009 | 0.066 | 0.053 |
| Active in 2010 (ref. Inactive) | 5.074^***^ | 4.994–5.154 | 0.895 | < 0.001 |

*Note*. OR = odds ratio; CI = confidential interval; GDS = Geriatric Depression Scale; IADL = instrumental activities of daily living. Missing values in covariates were imputed by stochastic regressions. ** *p* < 0.01; *** *p* < 0.001.
