# Supplementary material for: Types of social networks and starting leisure activities in later life: A longitudinal Japan Gerontological Evaluation Study (JAGES)
Source: PLoS One. 2021 Jul 15;16(7):e0254828. doi: 10.1371/journal.pone.0254828 (PMC8282000; doi:10.1371/journal.pone.0254828)
Supplement: S4 Table — (DOCX) [file pone.0254828.s004.docx]

**S4 Table. Result of the logistic regression analysis predicting the likelihood of resuming specific leisure activities among participants who had hobbies both in 2010 and 2013 (outcome: 1 = resuming activities; 0 = starting new activities)**

|  | OR | 95% CI | Cohen’s *d* | *p* value |
| --- | --- | --- | --- | --- |
| Latent class | | | | |
| Diverse (*n*=16, 9/7) | 0.608 | 0.205–1.808 | 0.274 | 0.371 |
| Same-Interest  (*n*=113, 89/24) | 1.933^*^ | 1.062–3.520 | 0.363 | 0.031 |
| Neighbor  (*n*=213, 148/65) | 1.261 | 0.782–2.034 | 0.128 | 0.342 |
| Colleague  (*n*=89, 57/32) | 0.915 | 0.516–1.622 | 0.049 | 0.760 |
| Covariate | | | | |
| Age (continuous) | 1.000 | 0.964–1.037 | 0.000 | 0.995 |
| Gender (ref. male) | 0.770 | 0.522–1.135 | 0.144 | 0.187 |
| Income (continuous in units of one million yen) | 1.138 | 0.980–1.321 | 0.071 | 0.091 |
| Education years ≥ 10  (ref. < 10) | 1.492^*^ | 1.009–2.205 | 0.221 | 0.045 |
| GDS ≥ 5 (ref. < 5) | 1.083 | 0.740–1.586 | 0.044 | 0.681 |
| IADL < 5 (ref. = 5) | 0.917 | 0.609–1.382 | 0.048 | 0.679 |
| Living alone (ref. Living with at least one family member) | 0.792 | 0.433–1.450 | 0.129 | 0.451 |

*Note*. Restricted Network (*n* = 141, 46/95) is the reference class. This analysis was conducted after assigning participants to their most likely latent class. The first element in parentheses shown after each latent class indicates the number of participants who had hobbies both in 2010 and 2013. The numerator (respectively, denominator) of the second element is the number of participants resuming the same activities as in 2010 (respectively, starting new activities).

OR = odds ratio; CI = confidential interval; GDS = Geriatric Depression Scale; IADL = instrumental activities of daily living. * *p* < 0.05.
